# Multi-scale prototype convolutional network for few-shot semantic segmentation

**DOI:** 10.1371/journal.pone.0319905

**Published:** 2025-04-15

**Authors:** Ding Xu, Shun Yu, Jingxuan Zhou, Fusen Guo, Lin Li, Jishizhan Chen

**Affiliations:** 1 Computer Science Department, Harbin Institute of Technology, Harbin, China; 2 School of Systems and Computing, University of New South Wales, Canberra, Australia; 3 Faculty of Science and Engineering, Southern Cross University, Gold Coast, Australia; 4 Mechanical Engineering, University College London, London, United Kingdom; Nanjing University of Science and Technology, CHINA

## Abstract

Few-shot semantic segmentation aims to accurately segment objects from a limited amount of annotated data, a task complicated by intra-class variations and prototype representation challenges. To address these issues, we propose the Multi-Scale Prototype Convolutional Network (MPCN). Our approach introduces a Prior Mask Generation (PMG) module, which employs dynamic kernels of varying sizes to capture multi-scale object features. This enhances the interaction between support and query features, thereby improving segmentation accuracy. Additionally, we present a Multi-Scale Prototype Extraction (MPE) module to overcome the limitations of MAP (Mean Average Precision). By augmenting support set features, assessing spatial importance, and utilizing multi-scale downsampling, we obtain a more accurate prototype set. Extensive experiments conducted on the PASCAL-$5^{i}$ and COCO-$20^{i}$ datasets demonstrate that our method achieves superior performance in both 1-shot and 5-shot settings.

## 1 Introduction

Semantic segmentation is a fundamental and critical task in computer vision, involving the assignment of a distinct class label to each pixel within an image. This pixel-level classification provides a comprehensive interpretation of visual data, enabling machines to perceive and understand the objects within an image with high spatial accuracy. Semantic segmentation plays a pivotal role in various practical applications, such as autonomous driving, medical image analysis, and satellite imagery, where precise object delineation is essential for decision-making and further analysis. However, traditional semantic segmentation methods rely heavily on large, labeled datasets to effectively train deep learning models. These datasets require meticulous annotation to ensure that the model can accurately differentiate between various object classes. The process of collecting and labeling such vast amounts of data is resource-intensive, costly, and time-consuming. Furthermore, the complexity and variability of real-world objects, along with the diverse range of image contexts, exacerbate the challenges associated with this task [1–3].

In recent years, few-shot segmentation has emerged as a promising solution to address the limitations of traditional segmentation approaches. Few-shot segmentation aims to mitigate the scarcity of labeled data by leveraging techniques from meta-learning and few-shot learning, enabling models to generalize from a limited number of annotated examples [4–6]. This capability is particularly crucial for applications that require rapid adaptation to new, previously unseen object classes or environments. However, few-shot semantic segmentation faces significant challenges, primarily due to the limited availability of labeled data for novel categories, compounded by substantial intra-class variability and inter-class similarity among segmentation targets.

Currently, the majority of Few-Shot Segmentation (FSS) methods predominantly rely on prototype-based approaches [7,8,10,11]. These methods extract segmentation cues by applying Masked Average Pooling (MAP) to the features from the support set. The prototypes generated through this process represent typical feature samples of the target objects in the support set images, and these prototypes are subsequently used to make predictions for the objects in the query image via techniques such as cosine similarity or feature concatenation. However, the simple averaging operation used in MAP has notable limitations. Specifically, it fails to preserve the diversity information and intrinsic object details captured by the individual pixels in the support images. By averaging the features, this process smooths out fine-grained object details, resulting in the loss of critical spatial and structural information necessary for accurate segmentation. As a result, the prototypes generated through MAP may lack sufficient discriminative power, significantly undermining segmentation accuracy, particularly when dealing with complex and diverse objects. Additionally, significant scale variations and appearance changes within object classes further exacerbate the challenge, leading to coarse segmentation results that lack precision and fine detail.

To tackle these challenges, we introduce a novel framework: the Multi-Scale Prototype Convolutional Network (MSPCNet). This framework is specifically designed to mitigate intra-class variation while effectively capturing the intrinsic details of objects. A pivotal component of our approach is the Prior Mask Generation Module (PMG), which enhances interactions between the features of the support set and the query image. By generating prior masks, the PMG facilitates a more effective alignment of support and query features, guiding the segmentation process and enabling the model to focus on the most relevant regions of the query image. Specifically, we employ three dynamic kernels, each with a different sliding window size. These kernels are strategically designed to extract features at varying spatial resolutions, enabling the model to capture both fine-grained details and large-scale object characteristics. The outputs from these kernels are subsequently utilized to generate distinct query activation maps, which guide the model’s attention to the diverse scales and appearances of objects in the query image. This multi-scale interaction strategy, though conceptually simple, is crucial for achieving robust segmentation. It allows the model to adapt effectively to the wide range of object sizes and structural variations within the query image, while preserving their intrinsic details, thereby enhancing both segmentation accuracy and robustness.

Additionally, to address the issue that Masked Average Pooling fails to adequately extract segmentation priors from support images, we propose a Multi-Scale Prototype Extraction Module (MPE). Specifically, we enhance support set features using a designed feature enhancement module and evaluate the importance of each spatial position vector. We then obtain a multi-scale feature set by applying different down-sampling factors and aggregate the support set masks with the multi-scale features to derive the prototype set. This prototype set, along with the query set features, is used to generate category-aware features. Finally, we fuse the category-aware features, query activation maps, and intermediate query features to produce refined query pseudo-masks. Our main contributions are as follows:

- We introduce the Prior Mask Generation (PMG) module, which captures multi-scale object features using dynamic kernels with varying sliding window sizes, thereby generating diverse query activation maps.
- We also introduce the Multi-Scale Prototype Extraction (MPE) module, which addresses the limitations of Mean Average Precision (MAP) by enhancing support set features, assessing spatial importance, and employing multi-scale downsampling.
- Extensive experiments conducted on PASCAL-$5^{i}$ and COCO-$20^{i}$ datasets demonstrate that our proposed Multi-Scale Prototype Convolutional Network (MPCN) achieves significant performance improvements compared to current methods.

## 2 Related work

Semantic segmentation is a vital area of research in computer vision, concentrating on the task of assigning class labels to each pixel in an image for a comprehensive understanding of visual content. Few-shot segmentation seeks to tackle the challenges of image segmentation with only a limited set of labeled examples, offering a distinctive opportunity for image analysis and interpretation using minimal training data.

### 2.1 Semantic segmentation

Semantic segmentation aims to provide a comprehensive understanding of visual scenes and precise object localization. Early approaches, such as Fully Convolutional Networks (FCNs) [12], pioneered the development of end-to-end networks tailored specifically for semantic segmentation tasks. These networks utilized dense forward computation and backpropagation to generate outputs that match the input image size, effectively allowing for pixel-wise classification. By integrating semantic information from various convolutional layers, FCNs improved the accuracy of semantic segmentation networks significantly.

As the field evolved, more sophisticated architectures emerged. Encoder-decoder frameworks and dilated convolution techniques, exemplified by models like U-Net [13], SegNet [14], and the DeepLab series [15], made substantial strides in enhancing segmentation precision. These models not only excelled in detail restoration but also improved contextual understanding, which is crucial for accurately identifying and delineating objects within complex scenes. To address the inherent challenges posed by multi-scale targets—where objects may appear at various sizes—Pyramid Pooling Modules (PPM) [16] and Atrous Spatial Pyramid Pooling (ASPP) [17] were introduced. These techniques significantly bolster the extraction of global contextual features, enabling models to recognize and segment objects more effectively, regardless of their scale.

Simultaneously, attention mechanisms gained traction, including spatial attention and both position and channel attention modules [18–20]. These mechanisms are instrumental in enhancing the aggregation of long-range contextual information, thereby enriching the model’s ability to represent semantic information more robustly.

Despite these advancements, traditional semantic segmentation methods encounter significant challenges, particularly in scenarios where annotated data is scarce or when there is a need to adapt to new categories that were not included in the training dataset. This dependency on large-scale annotated datasets constrains the scalability and generalization capabilities of these models, prompting researchers to explore alternative strategies such as Few-Shot Segmentation (FSS). FSS techniques aim to bridge this gap by enabling effective segmentation with minimal labeled examples, thus broadening the applicability of semantic segmentation in real-world scenarios.

### 2.2 Few-shot segmentation

Few-shot segmentation overcomes the limitations of traditional methods by enabling segmentation of objects using a minimal number of annotated examples for each class. The primary goal is to generalise from a small support set (labelled examples) to accurately classify objects within a query image.

Recent years have seen substantial advancements in prototype-based semantic segmentation for few-shot images [21,22].The SG-One network [23] optimizes feature maps and segmentation loss by utilizing two branches of shared convolutional layers. It employs Masked Average Pooling to extract prototype features from support images, measuring the prototype distance between support and query images using cosine similarity. The PANet [24] framework leverages prototype-based metric learning and incorporates prototype alignment regularization to maximize the information derived from support images. ASGNet [25] introduces a superpixel-guided clustering approach, which extracts multiple prototypes from support images and reconstructs their feature maps through a novel assignment strategy, thereby enhancing semantic extraction and representation. PFENet [26] aggregates multi-scale information from input samples to capture global feature information, significantly improving the model’s predictive capability. To address information loss issues, HSNet [27] focuses on feature interrelationships, converting dense feature-related tensors into segmentation results through high-dimensional convolutions. Additionally, advancements in attention mechanisms have further improved few-shot segmentation models. DENet [28] introduces a novel attention module that enhances algorithm generalization by adjusting the weights of the metric classifier. MCE [39] enhances few-shot segmentation by capturing shared visual properties and learning inter-image dependencies, improving pixel-wise labeling of unseen classes with limited annotated images. BAM [29] optimizes prediction results through a fully supervised semantic segmentation model, discarding erroneous predictions and proposing a new model based on base classes. The methods mentioned above primarily rely on prototype-based approaches, where features are extracted and prototypes are generated using Masked Average Pooling (MAP). However, the averaging operation in MAP fails to preserve the details and diversity information in the support set images, leading to the loss of fine spatial and structural information. As a result, the generated prototypes lack sufficient discriminative power. These methods often lead to a reduction in segmentation accuracy when dealing with complex and highly variable objects, particularly in cases where there are significant changes in object scale and appearance, causing the segmentation results to lack fine-grained details.

To address the limitations of current Few-Shot Segmentation (FSS) methods, we propose the Multi-Scale Prototype Convolutional Network (MPCN). The MPCN enhances feature interaction between the support set and query images through the PMG module, utilizing dynamic kernels at multiple scales to capture fine details and large-scale object features, thereby improving segmentation accuracy and robustness. Subsequently, the MPE module is employed to strengthen the support set features, apply downsampling to generate multi-scale features, and refine prototypes to achieve better query segmentation, thus overcoming the limitations of Masked Average Pooling (MAP).

## 3 Our approach

### 3.1 Few-shot segmentation

The task of few-shot segmentation aims to address image segmentation challenges under conditions of limited annotated data. In a typical few-shot semantic segmentation dataset $D_{\text{data}}$, the data is divided into training samples $D_{\text{train}}$ and test samples $D_{\text{test}}$, with the classes in $D_{\text{train}}$ and $D_{\text{test}}$ being mutually exclusive, i.e., $C_{\text{train}}\cap C_{\text{test}}=\emptyset$. During the training phase, the few-shot semantic segmentation model is trained on a large set of annotated training samples $D_{\text{train}}$, under the guidance of the ground truth labels $M_{q}$ for query images. The goal is to construct a segmentation model *f* ( *S* , *Q* )  that can utilize *K* annotated images $I_{s}$ and their corresponding support set masks $M_{s}$ to perform semantic segmentation on query images $I_{q}$, producing predictions $M_{q1}$ that approximate the ground truth $M_{q}$ (where *S* and *Q* represent the support and query sets, respectively). Consequently, during the inference phase, the model is able to rapidly generalize to new class data $D_{\text{test}}$ using the limited annotated data, thereby alleviating the dependency on extensive labeled datasets.

### 3.2 Overview

**Fig 1:**
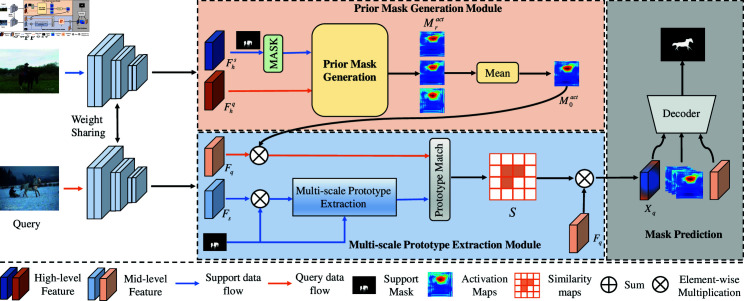
The overall architecture of our proposed Multi-Scale Prototype Convolutional Network (MPCN) for few-shot semantic segmentation is illustrated. Initially, high-level support and query features are fed into the Prior Mask Generation (PMG) module to generate the initial mask for the query image $M_{0}^{act}$, which is then used to perform foreground filtering on intermediate query set features. Subsequently, the Multi-Scale Prototype Extraction (MPE) module extracts support prototypes and matches these prototypes with the filtered query features to generate a similarity map and category-aware prototypes $X_{q}$. Finally, category-aware features, query activation maps, and intermediate query features are fused to produce a refined query pseudo-mask.

As illustrated in Fig 1, our Multi-Scale Prototype Convolutional Network comprises two key modules: the Prior Mask Generation (PMG) module and the Multi-Scale Prototype Extraction (MPE) module. Specifically, given the support and query images, $I_{s}$ and $I_{q}$, we employ a shared-weight backbone to extract mid-level and high-level features. The PMG module subsequently generates an initial mask $M_{0}^{act}$ for the target object in the query image and applies foreground filtering to the query set features. Subsequently, the MPE module extracts support prototypes and generates similarity map by matching these prototypes with the filtered query features. This process produces category-aware prototypes with contextual dependencies and rich semantics. During the decoder stage, we fuse the category-aware features, query activation maps, and intermediate query features to generate a refined query pseudo-mask. The technical specifics of each module will be addressed in the subsequent sections.

**Fig 2:**
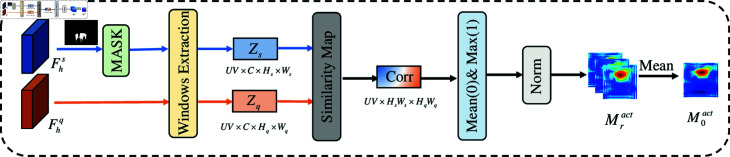
The specific structure diagram of Prior Mask Generation module.

### 3.3 Prior mask generation module

To align the features of the support set and query set, and to guide the segmentation model to focus on the most relevant regions in the query image, we introduce the PMG module. Specifically, we first use the high-level features from both the support set and the query set to generate a prior mask [8]. This prior mask, typically a matching between feature maps, represents an approximate location of the target object without considering the overall contextual relationships. To address this, we employ three sliding windows to achieve global and regional matching, as shown in Fig 2. Specifically, we utilize the high-level features from the support set and query set, denoted as $F_{s}^{h}\in {\mathbb{R}}^{C\times H_{s}\times W_{s}}$ and $F_{q}^{h}\in {\mathbb{R}}^{C\times H_{q}\times W_{q}}$, alongside binary masks $M_{s}\in {\mathbb{R}}^{C\times H_{s}\times W_{s}}$ as inputs. Here, *C* represents the channel dimension, while $H_{s},W_{s},H_{q},$ and $W_{q}$ denote the height and width of the support and query features, respectively. We then extract the regional features $R_{s}$ and $R_{q}$ using the designed sliding windows:


$$\begin{array}{ccc} R_{s}=W\left(F_{s}^{h}⊗M_{s}\right)\in {\mathbb{R}}^{UV\times C\times H_{s}W_{s}} & & \end{array}$$ (1)

$$\begin{array}{ccc} R_{q}=W\left(F_{q}^{h}\right)\in {\mathbb{R}}^{UV\times C\times H_{q}W_{q}} & & \end{array}$$ (2)

where  ⊗  denotes the Hadamard product, *W* represents the sliding window operation, and *U* , *V* are the height and width of the windows. We use three multi-scale windows, specifically (1,1), (3,3), and (5,5), to capture features corresponding to small, medium, and large objects. We then compute the cross-correlation features $D_{sq}$ based on similarity of $R_{s}$ and $R_{q}$:


$$\begin{array}{ccc} D=\text{Ein}(\prime icj,ick\to ijk^{\prime },R_{s},R_{q})\in {\mathbb{R}}^{UV\times H_{s}W_{s}\times H_{q}W_{q}} & & \end{array}$$ (3)

where *Ein* denotes the Einstein summation convention [9], *icj* represents the index of $R_{s}$, and *ick* represents the index of $R_{q}$; the final cross-correlation feature $D_{sq}$ has the index *ijk*, which indicates summation over index *c*. Finally, we average and normalize the cross-correlation features $D_{sq}$ to obtain the activation maps for the target object masks. Given that we use three different windows to obtain $M_{1}^{act}$, $M_{2}^{act}$, and $M_{3}^{act}$, we average these to produce $M_{0}^{act}$, which represents the approximate location of the target object in the query image.

**Fig 3:**
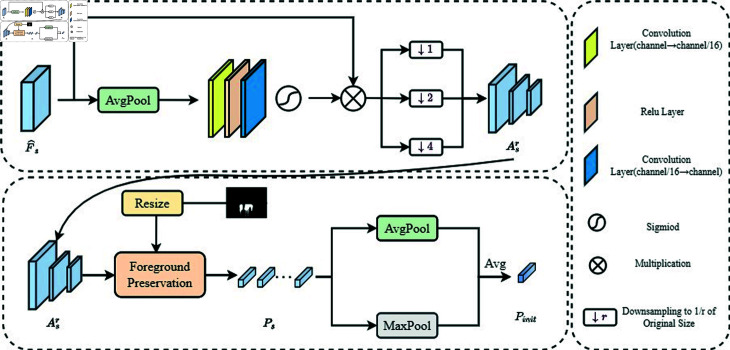
The specific structure diagram of Multi-Scale Prototype Extraction module.

### 3.4 Multi-scale prototype extraction module

The initial mask from the previous section only provides an approximate location of the target object. To capture more details, we construct a Multi-Scale Prototype Extraction (MPE) module to generate sufficiently informative and scale-sensitive support prototypes, and use a similarity spectrum module to filter out background from the query features, retaining the foreground to produce the final query class semantic feature $X_{q}$. Specifically, to mitigate interference from irrelevant classes, we perform a pixel-wise multiplication between the initial mask $M_{0}^{act}$ and the query image features $F_{q}$ to obtain ${\hat{F}}_{q}$. Similarly, we multiply the support mask $M_{s}$ with the support image features $F_{s}$ to obtain ${\hat{F}}_{s}$. The MS-CPE module then extracts rich semantic information from ${\hat{F}}_{s}$, as shown in Fig 3.

First, we apply average pooling to ${\hat{F}}_{s}$, followed by convolutional layers and activation functions to obtain the semantic-enhanced features $A_{s}$:


$$\begin{array}{ccc} A_{s}=\sigma \left(F_{\text{conv}}\left(A{\hat{F}}_{s}\right)\right) & & \end{array}$$ (4)

where $A{\hat{F}}_{s}$ denotes the support features after average pooling, $F_{\text{conv}}$ represents the convolutional layers and a rule layer, and *σ* is the Sigmoid function. To address the challenge of scale diversity within class samples, we obtain multi-scale feature sets $A_{s}^{r}$ by applying different down-sampling factors *r* ∈ { 1 , 2 , 4 } . Next, using $M_{s}^{r}$, we retain the foreground features in $A_{s}^{r}$ and flatten them into a set of feature vectors:


$$\begin{array}{ccc} P_{s}=\left\{A_{s}^{r}(x,y)I\left(M_{s}^{r}(x,y)=1\right)\right\} & & \end{array}$$ (5)

where  ( *x* , *y* )  denotes spatial positions, *I* is the indicator function that outputs 1 if the input condition is met and 0 otherwise, and $P_{s}$ represents the set of foreground feature vectors derived from the support image. Unlike methods that use direct mask average pooling, we use average pooling to extract general features, maximum pooling to retain significant features, and average the two pooled vectors to obtain the initial support prototype $P_{\text{init}}\in {\mathbb{R}}^{1\times C}$:


$$\begin{array}{ccc} P_{\text{init}}=\mathrm{Average}\left(\text{Avg}\left(P_{s}\right),\text{Max}\left(P_{s}\right)\right) & & \end{array}$$ (6)

where Avg and Max denote average pooling and maximum pooling, respectively. $P_{\text{init}}$ effectively summarizes the class semantic information. To achieve pixel-level predictions, we use a prototype matching model to construct a non-parametric similarity measure across spatial locations.

First, we calculate the cosine similarity between the feature vector ${\hat{F}}_{q}$ at each spatial location and the support prototype $P_{\text{init}}$. The cosine similarity is used as the exponent in an exponential function to obtain the similarity spectrum *S*:


$$\begin{array}{ccc} S(x,y)=\exp\left(\cos\text{-simi}\left(P_{\text{init}},{\hat{F}}_{q}(x,y)\right)\right) & & \end{array}$$ (7)

where *exp* ⁡   and *cos* ⁡  -simi denote the exponential function and cosine similarity, respectively;  ( *x* , *y* )  represents the spatial location; ${\hat{F}}_{q}\in {\mathbb{R}}^{1\times C}$ denotes the feature vector of ${\hat{F}}_{q}$ at the location  ( *x* , *y* ) ; and *S* ( *x* , *y* )  represents the similarity score between ${\hat{F}}_{q}(x,y)$ and $P_{\text{init}}$. We use the similarity spectrum *S* to reweight the query image features $F_{q}$, enhancing the foreground regions while suppressing the background, to obtain the category-aware feature $X_{q}$:


$$\begin{array}{ccc} X_{q}=S⊗F_{q} & & \end{array}$$ (8)

Finally, the category-aware feature $X_{q}$, the query activation maps ${\left\{M_{i}^{act}\right\}}_{i=0}^{3}$, and the intermediate query features $F_{q}$ are reshaped to the same spatial dimensions and fused together to produce the output feature $X_{\text{out}}\in {\mathbb{R}}^{2C+4\times H\times W}$:


$$\begin{array}{ccc} X_{\text{out}}=F_{\text{cat}}\left(X_{q},{\left\{M_{i}^{act}\right\}}_{i=0}^{3},F_{q}\right) & & \end{array}$$ (9)

where $F_{\text{cat}}$ denotes concatenation along the channel dimension. The final output $X_{\text{out}}$ is then input to the decoder to generate the segmentation mask ${\hat{M}}_{q}$ for the query image $I_{q}$:


$$\begin{array}{ccc} {\hat{M}}_{q}=F_{\text{cls}}\left(F_{\text{ASPP}}\left(F_{\text{conv}}\left(X_{\text{out}}\right)\right)\right) & & \end{array}$$ (10)

where $F_{\text{conv}}$, $F_{\text{ASPP}}$, and $F_{\text{cls}}$ are the consecutive modules constituting the decoder.

### 3.5 Training loss

Inspired by previous work [8,30,31], we employ the Binary Cross-Entropy (BCE) loss between the predicted mask ${\hat{M}}_{q}$ of the query image $I_{q}$ and the ground truth mask $M_{q}$ as the primary loss function for our model:


$$\begin{array}{ccc} L_{\text{seg}}=\frac{1}{hw}\overset{h}{\underset{i=1}{\sum }}\overset{w}{\underset{j=1}{\sum }}\text{BCE}\left({\hat{M}}_{q}(i,j),M_{q}(i,j)\right) & & \end{array}$$ (11)

Additionally, to ensure that the multi-scale contextual prototype extraction module generates more accurate support prototypes, we introduce an auxiliary loss $L_{\text{aux}}$. This auxiliary loss is computed by predicting the support mask ${\hat{M}}_{s}$ using the corresponding query prediction mask ${\hat{M}}_{q}$, with the generation of ${\hat{M}}_{s}$ being similar to the method described in Equation (10):


$$\begin{array}{ccc} L_{\text{aux}}=\frac{1}{hw}\overset{h}{\underset{i=1}{\sum }}\overset{w}{\underset{j=1}{\sum }}\text{BCE}\left({\hat{M}}_{s}(i,j),M_{s}(i,j)\right) & & \end{array}$$ (12)

Here, $L_{\text{aux}}$ represents the BCE loss between $M_{s}$ and ${\hat{M}}_{s}$. Thus, the final loss function is:


$$\begin{array}{ccc} L=L_{\text{seg}}+\lambda L_{\text{aux}} & & \end{array}$$ (13)

where *λ* is a balancing factor between the segmentation loss $L_{\text{seg}}$ and the auxiliary loss $L_{\text{aux}}$. In our experiments, *λ* is set to 1.0.

## 4 Experiments

### 4.1 Dataset

PASCAL-$5^{i}$ [32] is a few-shot segmentation variant derived from the PASCAL VOC dataset [33], designed to evaluate the performance of few-shot learning methods. It divides the 20 object categories covered by the PASCAL dataset into 4 subsets, with each subset containing 5 classes for testing and the remaining 15 classes for training.

COCO-$20^{i}$ [34] is a variant of the COCO dataset [35] specifically designed for few-shot segmentation tasks. It partitions the 80 object categories in the COCO dataset into 4 subsets, with each subset containing 20 classes for testing and the remaining 60 classes for training.

### 4.2 Experimental settings

**Metrics and Evaluation.** Consistent with previous work, we utilize mean intersection over union (mIoU) and foreground-background IoU (FB-IoU) as our evaluation metrics. mIoU is a widely accepted standard for assessing segmentation performance, as it computes the ratio of the intersection to the union of predicted and ground truth regions for each category, averaging the results across all categories to gauge overall performance.


$$\begin{array}{ccc} mIoU=\frac{1}{N}\overset{N}{\underset{i=1}{\sum }}\frac{\left|Y_{i}\cap {Ŷ}_{i}\right|}{\left|Y_{i}\cup {Ŷ}_{i}\right|}, & & \end{array}$$ (14)

where *N* is the number of categories, $Y_{i}$ is the ground truth for category *i*, and ${Ŷ}_{i}$ is the prediction for category *i*. Moreover, FB-IoU offers a comprehensive assessment of the model’s segmentation capabilities by calculating the IoU between the predicted foreground and background regions and their corresponding ground truth segments. This metric provides a more precise evaluation of the model’s performance in managing complex scenes.


$$\begin{array}{ccc} FB-IoU=\frac{\left|Y_{f}\cap {Ŷ}_{f}|+|Y_{b}\cap {Ŷ}_{b}\right|}{\left|Y_{f}\cup {Ŷ}_{f}|+|Y_{b}\cup {Ŷ}_{b}\right|}, & & \end{array}$$ (15)

where $Y_{f}$ and $Y_{b}$ represent the foreground and background in the ground truth, respectively, and ${Ŷ}_{f}$ and ${Ŷ}_{b}$ are the corresponding predictions for the foreground and background.

**Implementation Details.** In our few-shot segmentation experiments, we selected VGG-16 [36] and ResNet-50 [37] as backbone networks, both pretrained on the ImageNet classification task, with their weights fixed during training. The implementation was carried out using PyTorch 1.9.0, and experiments were executed on an NVIDIA RTX 3090 GPU. All images were cropped to a size of 473 × 473 pixels for training. We utilized stochastic gradient descent (SGD) for optimization, setting the initial learning rate to 0.005, a batch size of 8, weight decay to 0.0001, and momentum to 0.9. Within the PMG module, sliding window sizes were configured to (1,1), (3,3), and (5,5). For the MPE module, downsampling factors *r* ∈ { 1 , 2 , 4 }  were employed. Training was conducted for 200 epochs on the PASCAL-5*i* dataset and for 50 epochs on the COCO-20*i* dataset.

### 4.3 Comparison with state-of-the-art methods

**PASCAL-$5^{i}$**: Table 1 provides a comparison of our model, MPCN, with various state-of-the-art methods on the PASCAL-$5^{i}$ dataset. Using the VGG16 backbone, MPCN surpasses most of the evaluated methods, attaining 63.2% mIoU and 75.6% FB-IoU in the 1-shot setting, and 68.6% mIoU and 79.1% FB-IoU in the 5-shot setting. Likewise, with the ResNet50 backbone, MPCN exhibits notable improvements. When compared to the most recent methods, SiGCN and MCE, MPCN achieves the highest mIoU and FB-IoU scores with the ResNet50 backbone. In the 1-shot setting, MPCN enhances mIoU by 2.3% compared to SiGCN and by 1.7% relative to MCE. In the 5-shot setting, MPCN increases mIoU by 2.6% compared to SiGCN and by 1.1% compared to MCE. Notably, MPCN also showed the highest foreground-to-background intersection ratio (FB-IoU) value, further emphasizing its effectiveness in distinguishing between foreground and background.

**Table 1:** The performance of the metrics on PASCAL-$5^{i}$ was evaluated using mean Intersection over Union (mIoU) (%) and Foreground-Background Intersection over Union (FB-IoU) (%). Results are presented with bold text indicating the best performance. The baseline results represent performance achieved without incorporating any additional modules (e.g., PMG and MPE).

Methods	Backbone	1-shot						5-shot					
		Fold-0	Fold-1	Fold-2	Fold-3	Mean	FB-IoU	Fold-0	Fold-1	Fold-2	Fold-3	Mean	FB-IoU
PFENet [26]	VGG16	56.9	68.2	54.4	52.4	58.0	72.0	59.0	69.1	54.8	52.9	59.0	72.3
MMNet [38]	VGG16	57.1	67.2	56.6	52.3	58.3	-	56.6	66.7	63.6	56.5	58.3	-
NTRENet [31]	VGG16	57.7	67.6	57.1	53.7	59.0	-	60.3	68.0	55.2	57.1	60.2	-
HSNet [27]	VGG16	59.6	65.7	59.6	54.0	59.7	73.4	64.9	69.0	64.1	58.6	64.1	76.6
BAM [29]	VGG16	59.9	67.5	64.9	55.7	62.0	-	64.0	71.5	69.4	63.6	67.1	-
MCE [39]	VGG16	60.6	69.5	65.1	56.3	62.9	74.5	65.6	72.8	69.7	64.7	68.2	78.2
CANet [40]	ResNet50	52.5	65.9	51.3	51.9	55.4	-	55.5	67.8	51.9	53.2	57.1	-
ReRPI [41]	ResNet50	59.8	68.3	62.1	48.5	59.7	-	64.6	71.4	71.1	59.3	66.6	-
SSP [42]	ResNet50	60.5	67.8	66.4	51.0	61.4	-	67.5	72.3	**75.2**	62.1	69.3	-
PFENet [26]	ResNet50	61.7	69.5	55.4	56.3	60.8	73.3	63.1	70.7	55.8	57.9	61.9	73.9
SCL [43]	ResNet50	63.0	70.0	56.5	57.7	61.8	71.9	64.5	70.9	57.3	58.7	62.9	72.8
SiGCN [44]	ResNet50	65.1	70.1	65.2	60.8	65.3	77.5	68.9	72.6	66.8	65.8	68.5	78.3
MCE [39]	ResNet50	65.3	71.2	66.2	61.0	65.9	78.1	69.2	73.7	70.5	66.8	70.0	81.3
Baseline	VGG16	56.9	65.2	63.6	52.4	58.5	71.8	63.1	68.4	66.3	61.8	64.9	75.7
MPCN	VGG16	61.3	70.1	64.8	56.7	63.2	75.6	66.4	73.5	69.2	65.4	68.6	79.1
Baseline	ResNet50	63.3	68.7	64.1	57.4	63.4	74.6	66.4	70.7	67.2	63.1	66.9	77.2
MPCN	ResNet50	**66.9**	**73.2**	**68.4**	**61.7**	**67.6**	**78.7**	**70.2**	**74.5**	71.4	**68.1**	**71.1**	**82.5**

**Table 2:** The performance of the metrics on COCO-$20^{i}$ was evaluated using mean Intersection over Union (mIoU) (%) and Foreground-Background Intersection over Union (FB-IoU) (%). Results are presented with bold text indicating the best performance. The baseline results represent performance achieved without incorporating any additional modules (e.g., PMG and MPE).

Methods	Backbone	1-shot						5-shot					
		Fold-0	Fold-1	Fold-2	Fold-3	Mean	FB-IoU	Fold-0	Fold-1	Fold-2	Fold-3	Mean	FB-IoU
PFENet [26]	VGG16	33.4	36.0	34.1	32.8	34.1	60.0	36.0	40.7	38.1	36.1	37.7	61.6
DPCN [8]	VGG16	38.5	43.7	38.2	37.7	39.5	62.5	42.7	51.6	45.7	44.6	46.2	66.1
BAM [29]	VGG16	36.4	47.1	43.3	41.7	42.1	-	42.9	51.4	48.3	46.6	47.3	-
SSP [42]	ResNet50	35.5	39.6	37.9	36.7	37.4	-	40.6	47.0	45.1	43.9	44.1	-
HSNet [27]	ResNet50	36.3	43.1	38.7	38.7	39.2	68.2	43.3	51.3	48.2	45.0	46.9	70.7
NTRENet [31]	ResNet50	36.8	42.6	39.9	37.9	39.3	68.5	38.2	44.1	40.4	38.4	40.3	69.2
CyCTR [45]	ResNet50	38.9	43.0	39.6	39.8	40.3	-	41.1	48.9	45.2	47.0	45.6	-
SiGCN [44]	ResNet50	38.7	46.3	43.1	37.5	41.4	62.7	44.9	54.5	46.5	45.9	48.0	66.2
DPCN [8]	ResNet50	42.0	47.0	43.2	39.7	43.0	63.2	46.0	54.9	50.8	47.4	49.8	67.4
MCE [39]	ResNet50	42.1	48.3	43.7	42.8	44.2	-	47.8	55.2	50.8	50.3	51.0	-
Baseline	VGG16	36.9	41.2	37.8	37.2	38.3	60.2	39.1	48.4	42.1	41.8	42.9	63.7
MPCN	VGG16	40.4	45.6	42.2	41.4	42.4	64.5	43.8	52.3	47.8	47.4	47.8	67.8
Baseline	ResNet50	38.1	44.7	41.6	39.4	41.0	63.8	43.7	51.2	48.1	48.6	47.9	66.7
MPCN	ResNet50	**42.6**	**49.1**	**45.2**	**43.6**	**45.1**	**69.4**	**48.6**	**56.5**	**52.4**	**51.7**	**52.3**	**71.2**

**COCO-$20^{i}$**: COCO-$20^{i}$ presents a more challenging environment, featuring a greater number of categories and more complex scenes. Table 2 illustrates the performance of MPCN in comparison to other methods on the COCO-$20^{i}$ dataset. Notably, MPCN excels in both the 1-shot and 5-shot settings. Using the VGG16 backbone, MPCN achieves an average Intersection over Union (mIoU) of 42.4% in the 1-shot setting and 47.8% in the 5-shot setting, significantly outperforming other methods such as DPCN and BAM. When using the ResNet50 backbone, MPCN’s performance is even more pronounced. In the 1-shot setting, MPCN reaches an average mIoU of 45.1%, outperforming SiGCN and MCE by 3.7% and 0.9%, respectively. In the 5-shot setting, MPCN achieves an average mIoU of 52.3%, exceeding SiGCN and MCE by 4.3% and 1.3%, respectively. Additionally, MPCN excels in FB-IoU, with scores of 69.4% and 71.2% in the corresponding settings.

The Multi-Scale Prototype Convolutional Network (MPCN) addresses intra-class variability and prototype representation issues through the Prior Mask Generation (PMG) and Multi-Scale Prototype Extraction (MPE) modules, achieving significant improvements in segmentation performance on the PASCAL-$5^{i}$ and COCO-$20^{i}$ datasets. However, the use of multiple dynamic kernels with varying sliding window sizes and multi-scale feature extraction may lead to a substantial increase in computational cost and memory usage.

**Table 3:** Ablation studies of main model components.

id	PMG	MPE	Fold-0	Fold-1	Fold-2	Fold-3	Mean	FB-IoU
(a)			63.3	68.7	64.1	57.4	63.4	74.6
(b)	*✓*		64.5	70.2	66.4	59.1	65.1	76.3
(c)		*✓*	65.4	71.6	67.2	59.8	66.0	77.4
(d)	*✓*	*✓*	**66.9**	**73.2**	**68.4**	**61.7**	**67.6**	**78.7**

### 4.4 Ablations

To assess the impact of various modules on the model’s performance, we conducted an ablation study utilizing the ResNet-50 backbone in the 1-shot setting on the PASCAL-$5^{i}$ dataset. As presented in Table 3, the baseline model, which excludes the two modules (configuration (a)), achieves an mIoU of 63.4% and an FB-IoU of 74.6%. Introducing only the PMG module (configuration (b)) increases the average mIoU to 65.1% and the average FB-IoU to 76.3%. Adding only the MPE module (configuration (c)) further improves the average mIoU to 66.0% and the FB-IoU to 77.4%. The most notable performance enhancement occurs when both the PMG and MPE modules are utilized together (configuration (d)), resulting in a 4.2% increase in mIoU and a 4.1% rise in FB-IoU compared to the baseline model. The ablation study results indicate that both the PMG and MPE modules significantly enhance the performance of the MPCN model. While each module individually contributes to improving the mIoU and FB-IoU metrics, their combined application yields the highest performance.

### 4.5 Impact of hyperparameters and comparison with other refinementmethods

In this subsection, we provide a detailed explanation of the selection of key hyperparameters, as well as a comparison with other refinement methods to highlight the advantages of the MPE module.

**Fig 4:**
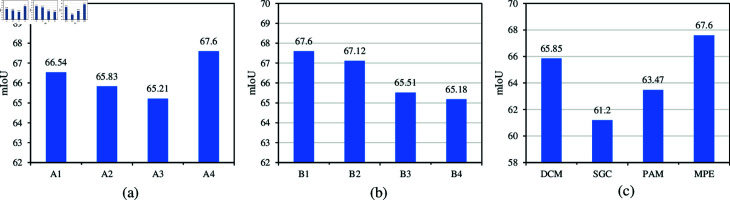
Figure (a) investigates the impact of different sliding window sizes on the model’s performance. Figure (b) explores the effect of various downsampling factor combinations on the model’s performance. Figure (c) presents a comparison of MPE with other refinement methods. All experiments were conducted under the PASCAL-$5^{i}$ 1-shot setting.

Fig 4a investigates the impact of different sliding window sizes on model performance. A1 represents the use of a sliding window of size (3,3), A2 represents a sliding window of size (5,5), A3 represents a sliding window of size (7,7), and A4 corresponds to the original configuration with sliding windows of sizes (1,1), (3,3), and (5,5). As shown in Fig 4a, the A4 configuration achieves the optimal result, demonstrating that the multi-scale sliding window setting significantly enhances the model’s performance.

Fig 4b explores the impact of different downsampling factor combinations on model performance. In Fig 4b, B1 represents the use of downsampling factors [1,2,4], B2 uses [1,4,4], B3 uses [1,4,8], and B4 employs [1,8,8]. As observed from Fig 4b, the B1 configuration yields the best performance, indicating that this downsampling factor setting allows the model to effectively capture the key features of the data, thus improving performance.

Additionally, Fig 4c shows a comparison between MPE and other refinement methods. Here, DCM [8] represents dynamic convolution techniques, SGC [46] refers to superpixel-guided clustering, and PAM [47] corresponds to prototype activation methods. As seen in Fig 4c, our method achieves the best mIoU results. Compared to dynamic and weighted adaptive clustering techniques such as DCM, SGC, and PAM, the MPE module significantly improves segmentation accuracy. By leveraging dynamic kernels for multi-scale feature extraction, our approach not only enhances feature alignment but also adapts more effectively to variations in object size and structure.

### 4.6 Qualitative results

**Fig 5:**
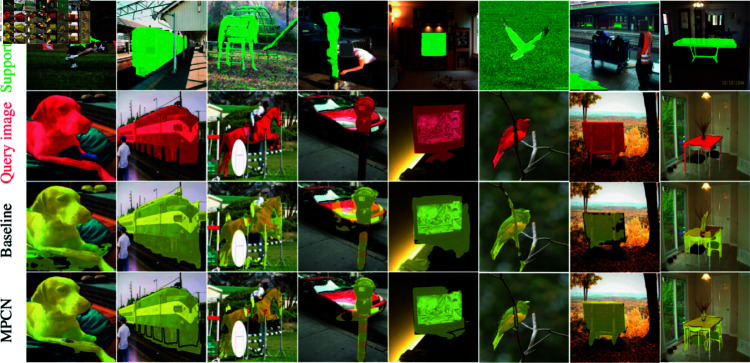
Results of our approach MPCN and other model on PASCAL-$5^{i}$ and COCO-$20^{i}$ datasets.

Fig 5 illustrates the segmentation results of MPCN in comparison to the baseline model on the PASCAL-$5^{i}$ and COCO-$20^{i}$ datasets. As depicted, MPCN demonstrates greater accuracy in segmenting target objects and captures finer details more effectively than the baseline model. Specifically, in columns 1, 3, 4, and 6 of Fig 5, our proposed MPCN accurately segments the target objects, whereas the baseline model incorrectly includes irrelevant background elements in the segmentation.

**Fig 6:**
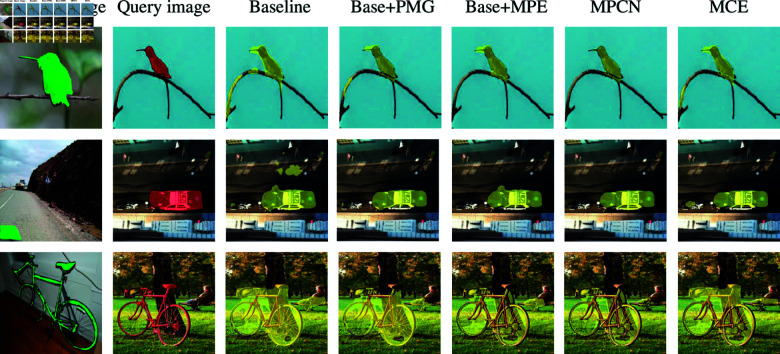
Visualization of different ablative results and compare it with other methods on PASCAL-$5^{i}$ and COCO-$20^{i}$ datasets.

As shown in Fig 6 we Visualization of different ablative results on the PASCAL-$5^{i}$ and COCO-$20^{i}$ datasets and compare the comparative results of the other methods. It can be seen that by adding PMG and MPG modules to the baseline model, the network can filter out irrelevant background regions and discover more target parts. For example, adding the fifth line of MPG removes the redundancy of the segmentation of birds and cars compared to the base prediction. In addition, adding the MPG module results in a more refined segmentation. For example, compared to the base prediction, the sixth row can filter out a large number of irrelevant background regions and localize the target object accurately. Finally, we compare our method with MCE, and as shown in Fig 6, our approach accurately segments the bird and the car. In contrast, MCE erroneously includes the nearby motorcycle in its vehicle segmentation and fails to fully capture the car as a whole.

## 5 Conclusion

In this study, we tackle the challenges of few-shot semantic segmentation by proposing the Multi-Scale Prototype Convolutional Network (MPCN), which incorporates two innovative modules: Prior Mask Generation (PMG) and Multi-Scale Prototype Extraction (MPE). Our approach effectively addresses intra-class variability and prototype representation issues by utilizing dynamic kernels of varying sizes to capture multi-scale features and enhance feature interactions. The PMG module refines query predictions, thereby improving segmentation precision. Meanwhile, the MPE module addresses the limitations of traditional Masked Average Pooling by generating a more accurate prototype set through enhanced feature augmentation and multi-scale analysis. Extensive experiments conducted on the PASCAL-$5^{i}$ and COCO-$20^{i}$ datasets demonstrate that our method achieves substantial improvements in segmentation performance, surpassing existing techniques in both the 1-shot and 5-shot settings.
